# Gestational Tubal Choriocarcinoma Presenting as a Pregnancy of Unknown Location following Ovarian Induction

**DOI:** 10.1155/2018/4705192

**Published:** 2018-05-03

**Authors:** Lawrence Hsu Lin, Koji Fushida, Eliane Azeka Hase, Regina Schultz, Laysa Manatta Tenorio, Fabricia Andrea Rosa Madia, Evelin Aline Zanardo, Leslie Domenici Kulikowski, Rossana Pulcineli Vieira Francisco

**Affiliations:** ^1^University of Sao Paulo Trophoblastic Disease Center, University of Sao Paulo Medical School, Sao Paulo, SP, Brazil; ^2^Department of Pathology, University of Sao Paulo Medical School, Sao Paulo, SP, Brazil; ^3^Cytogenomic Laboratory, Department of Pathology, University of Sao Paulo Medical School, Sao Paulo, SP, Brazil

## Abstract

The management of pregnancy of unknown location (PUL) can be a challenging situation, since it can present as several different conditions. Here we describe a rare case of gestational choriocarcinoma arising in the fallopian tube after ovarian induction in an infertile patient. The patient received clomiphene for ovarian induction and had rising levels of human chorionic gonadotropin (hCG) over nine months without sign of pregnancy. After referral to our center, the patient was diagnosed with a paraovarian tumor, which revealed a gestational choriocarcinoma arising in the fallopian tube; the final diagnosis was supported by pathological and cytogenomic analysis. Malignancies, such as gestational trophoblastic disease, should be in the differential diagnosis of PUL; the early recognition of these conditions is key for the proper treatment and favorable outcome.

## 1. Introduction

Pregnancy of unknown location (PUL) is a condition that can be particularly challenging for clinicians due to the variety of diagnoses that PUL can represent [1]. PUL can occur via either spontaneous conception or assisted reproduction treatment (ART). In certain cases, the use of ART should raise concerns, since ART is an important risk factor for ectopic pregnancies, which are associated with high rates of life-threatening complications [2]. However, PUL can also be the initial presentation of a variety of human chorionic gonadotropin- (hCG-) secreting malignancies [3].

Here, we report a case of tubal choriocarcinoma that initially presented as PUL in an infertile patient after ovarian induction. The gestational origin of the tumor was confirmed via short tandem repeat (STR) analysis of samples from the tumor and serum samples from the patient and her partner.

## 2. Case Report

A 38-year-old nulliparous woman was referred to the University of Sao Paulo Trophoblastic Disease Center due to PUL with increasing hCG levels, amenorrhea for 9 months, and no sign of an hCG-producing site. She had a prior history of primary infertility for years and had received clomiphene for ovarian induction. Her hCG rose from an initial level of 2,845 mIU/mL to 3,917 mIU/mL after 2 days, 5,533 mIU/mL after two weeks, and 381,808 mIU/mL after 9 months, with serial normal ultrasound scans performed during follow-up at another institution.

**Figure 1:**
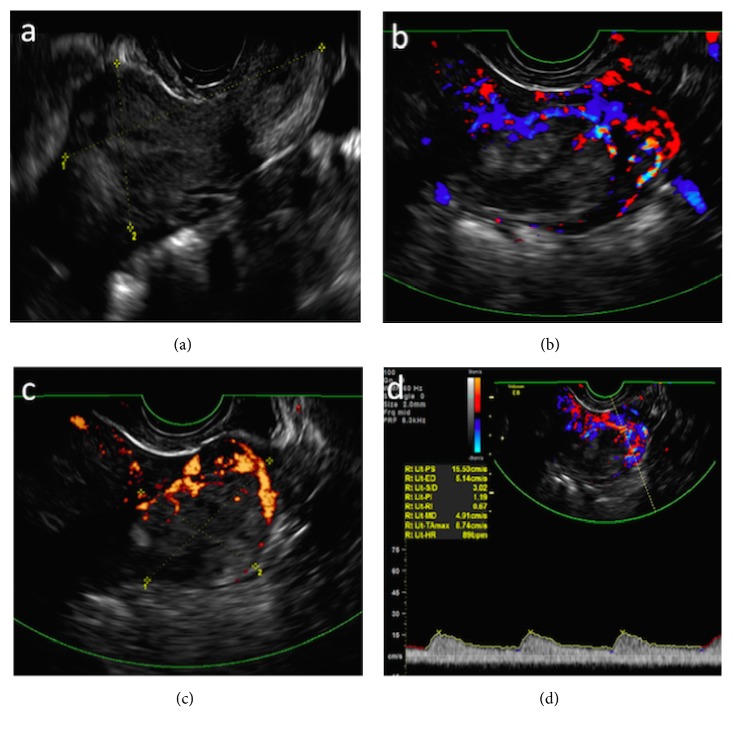
(a) Transvaginal sonographic sagittal section of the uterus revealing no signs of intrauterine pregnancy. (b) Color Doppler and (c) power Doppler transvaginal sonographic transverse sections of the left paraovarian tumor with strong peripheral vascularization. (d) Pulsed Doppler analysis of tumor vascularization, showing a pattern of low resistance.

**Figure 2:**
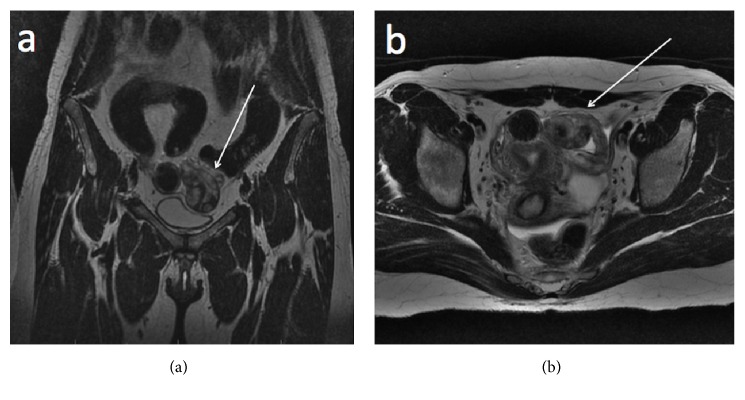
(a) T1-weighted coronal and (b) axial pelvic magnetic resonance imaging showing a cystic-solid lesion originating from the left fallopian tube (white arrow).

When the patient was referred to our institution, her hCG level was 267,836 mIU/mL, and ultrasound showed a normal uterus, a normal left ovary, a large cystic structure on the right ovary that measured 7.5 cm × 5.5 cm, and an irregular left paraovarian mass that measured 4.6 cm × 3.7 cm and exhibited intense low-resistance peripheral vascularization on Doppler examinations (Figures 1(a) and 1(b)). Pelvic magnetic resonance imaging was performed to further evaluate the origin of these findings; this imaging confirmed the presence of a solid-cystic lesion measuring 4.5 cm × 3.2 cm with a clear cleavage interface to the left ovary and postcontrast enhancement (Figures 2(a) and 2(b)). Brain, chest, and upper abdomen CT scans showed normal results.

**Figure 3:**
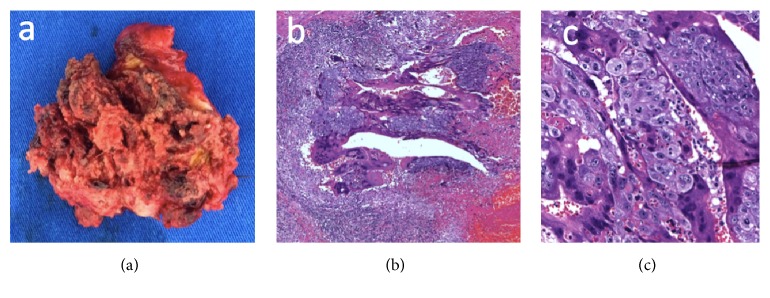
(a) Macroscopic appearance of the tumor. ((b) and (c)) Histological section of the tumor displaying clusters of abnormal syncytiotrophoblast and cytotrophoblast cells (hematoxylin-eosin staining, (b) ×50 magnification and (c) ×200 magnification).

An exploratory laparotomy was performed, resulting in visualization of a 5 cm vascularized left tubal mass, an 8 cm serous right ovarian cyst, and no other evidence of abdominal disease. Excision of the right ovarian cyst and the left uterine tube was performed. Pathological and immunohistochemical analyses revealed a choriocarcinoma infiltrating the tubal wall up to the serosa, the presence of vascular infiltration in tubal vessels, and a corpus luteum as the right ovarian cyst (Figure 3).

Also, in order to clarify the origin of the tumor we performed the differential diagnosis by genotyping seven autosomal STR loci (D13S317, D7S820, D2S1338, D21S11, D16S539, D18S51, CSF1PO, and FGA) and the sex-determining marker using AmpFLSTR® MiniFiler™ PCR Amplification Kit (Life Technologies™, California, USA) according to manufacturer's instructions.

**Figure 4:**
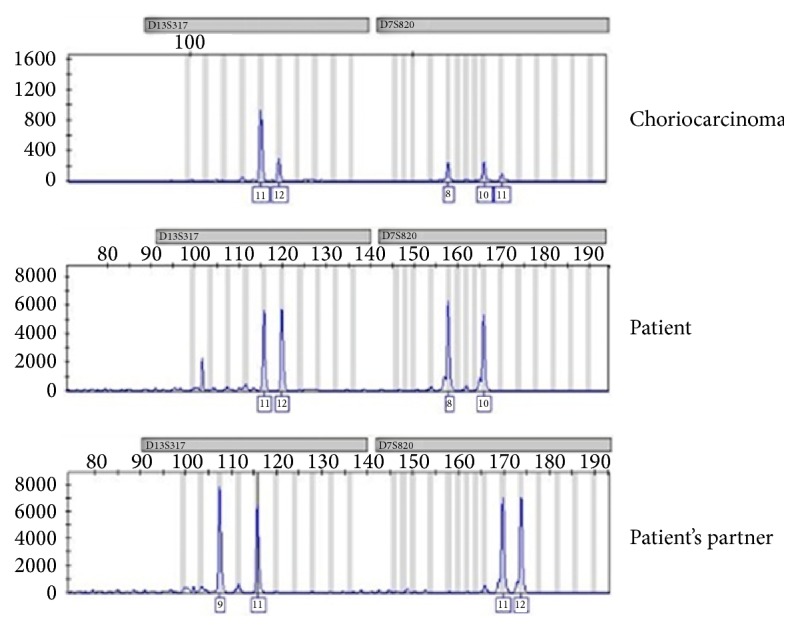
Results of genotyping of two autosomal STR loci (D13S317, D7S820) obtained from choriocarcinoma, patient, and patient's partner. The choriocarcinoma's electropherogram shows the presence of three allele for each STR loci. In the D13S317 presents the alleles 11 and 12, patient origin, and allele 11, patient's partner origin. The D7S820 presents the alleles 8 and 10, patient origin, and allele 11, patient's partner origin.

Cytogenomic analysis showed the presence of paternal alleles in choriocarcinoma tissue, confirming the gestational origin of the tumor (Figure 4).

The patient received 8 cycles of methotrexate, and her hCG levels normalized 4 months after surgery. The patient remains healthy 2 years after the completion of chemotherapy, with no signs of recurrence.

## 3. Discussion

PUL can be a challenging dilemma in medical practice, since several clinical entities can present with increased hCG levels and no visible sign of pregnancy [1, 2]. Early or failing intrauterine pregnancies, ectopic pregnancies, heterophile antibodies, and hCG-secreting tumors are examples of medical conditions that could initially present as PUL [2, 3]. Most guidelines suggest a diagnostic flow diagram based on levels and trends of hCG [17]. Increasing levels of hCG are more commonly associated with viable pregnancies than with other medical conditions; however, extremely high hCG values typically indicate a neoplastic process, particularly if no pregnancy is readily detectable.

Gestational trophoblastic disease (GTD) is a spectrum of disorders that arise from the placental trophoblast [18, 19]. One of the most aggressive types of GTD is gestational choriocarcinoma, which typically arises in the uterus. The presence of choriocarcinoma in the fallopian tube is extremely rare, with only four cases involving this phenomenon reported among 6,708 patients with GTD at Weston Park Hospital and six such cases among 2,100 cases of GTD at the New England Trophoblastic Disease Center [20, 21]. A tubal choriocarcinoma can be mistaken for an ectopic pregnancy due to the presence of an adnexal mass with raised hCG levels and can even present with tubal rupture and hemoperitoneum; therefore, pathological evaluation of tubal specimens is critical for appropriate differential diagnosis [11, 20]. In the case described here, besides presenting with very high hCG levels, the adnexal tumor showed peripheral low-resistance vascularization with an avascular central region (Figures 1(b), 1(c), and 1(d)), which resembles the compact pattern described by Hsieh et al. (1994), commonly associated with choriocarcinoma [22].  Table 1 summarizes the data from recently published cases of tubal choriocarcinoma in the literature, showing that most patients presented with symptoms that resemble ectopic pregnancies and higher hCG levels (median serum hCG: 15,000 mIU/mL; range: 3160–326,100 mIU/mL).

Since GTD is a rare condition, the relationship between ART and development of GTD has been debated in the literature. A retrospective report from United States of America disclosed a higher frequency of hydatidiform moles following ART (1 : 659 pregnancies) as compared to spontaneous pregnancies (estimated incidence 1 : 1000 pregnancies), even though it represents a rare complication [23, 24]. There seems to be a high percentage of multiple pregnancies with complete mole and coexisting fetus following ART, reaching 13% in a large retrospective cohort [24, 25]. However, a retrospective study in the United Kingdom found no statistical difference in the frequency of infertility treatment in patients with normal pregnancies and the ones with GTD [26].

ART is a risk factor for developing extrauterine pregnancies; therefore, ART may potentially increase the risk for gestational choriocarcinoma arising in unusual locations [10]. Other reports have described cases of tubal choriocarcinoma following ovarian induction with intrauterine insemination [10] and with in vitro fertilization [27]. However, data from the literature indicate that ART does not seem to influence the development of gestational trophoblastic neoplasia after hydatidiform moles [24, 26].

hCG is a key tumor marker in the management of patients with GTD because its levels are correlated with disease burden [18, 19]. In the presented case, the ectopic hCG-producing site was not initially detected using standard diagnostic methods, possibly because it was insufficiently large at first presentation. Since hCG is highly produced by choriocarcinoma cells, the same hCG level in a choriocarcinoma would reflect a much smaller mass of trophoblastic cells than of nonneoplastic trophoblasts, which are present in ectopic pregnancies [17, 18]. Most cases recently reported in the literature showed larger pelvic tumors, with a median size of 4 cm, ranging from 2 cm to 16 cm (Table 1).

Choriocarcinoma, particularly when presenting in unusual locations, can be of gestational or nongestational origin. STR analysis is a useful tool for determining tumor origin, which can impact treatment modalities and outcomes for patients with this tumor [28–30]. Gestational choriocarcinoma is highly sensitive to chemotherapy, as was observed for the patient described in this case report; in contrast, nongestational tumors are less sensitive to chemotherapy and demand more aggressive therapy because of worse outcomes [28, 29]. Since most centers do not have genetic analysis readily available (Table 1 shows that only 1 of 13 recently published cases of tubal choriocarcinoma reported genetic analysis of the tumor), differentiation between gestational and nongestation origin is based on clinical data, which is not always accurate, especially in trophoblastic tumors with unusual presentations [29].

In conclusion, differential diagnosis for PUL includes a variety of medical conditions. Early recognition of the hCG-producing source is key for the appropriate management of patients, particularly patients with neoplastic processes, which might be suspected based on extremely high and increasing levels of hCG combined with no signs of pregnancy.

## Figures and Tables

**Table 1:** Well-documented tubal choriocarcinoma case reports published in the literature in the last 10 years.

Study	Age	Last menstrual period	hCG levels (mIU/mL)	Clinical presentation	Tumor size (cm)	Surgical management	Chemotherapy	Genetic analysis
Bacalbasa et al., 2018 [4]	19	NA	NA	Abdominal pain and vaginal bleeding at presentation. Sigmoid colon invasion at recurrence	5	Unilateral salpingectomy at presentation. Recurrence managed with total hysterectomy, contralateral adnexectomy and sigmoid colon resection	MTX and ActD	No

Boynukalin et al., 2011 [5]	38	7 weeks	>15,000	Abdominal pain and vaginal bleeding	3.4	Unilateral salpingectomy	NA	NA

Butler et al., 2010 [6]	24	6 weeks	15,000	Abdominal pain and vaginal bleeding	3	Unilateral salpingectomy	MTX	No

Cianci et al., 2014 [7]	30	20 weeks	24,474	Coexisting intrauterine pregnancy and abdominal pain. Pulmonary metastasis	8	Unilateral adnexectomy at 20 weeks (delivery at 31 weeks)	EMA-CO after delivery	No

Davies et al., 2010 [8]	24	6 weeks	15,000	Abdominal pain and vaginal bleeding	3	Unilateral salpingectomy	MTX	No

Jia et al., 2017 [9]	39	6 weeks	7,158	Vaginal bleeding and palpable abdominal mass. Pulmonary metastasis	14	Total abdominal hysterectomy and bilateral adnexectomy	Yes (type not reported)	No

Jwa et al., 2017 [10]	34	6 weeks	7,054	Asymptomatic	2	Unilateral salpingectomy	EMA-CO	No

Karaman et al., 2015 [11]	31	7 weeks	29,251	Abdominal pain, fatigue, hypotension and tachycardia	4	Unilateral salpingectomy	MTX	No

Lin et al., 2017	38	9 months	267,836	Asymptomatic	4.6	Unilateral salpingectomy	MTX	Yes

Mehrotra et al., 2012 [12]	30	3.5 months	326,100	Abdominal pain, fever, fatigue, tachycardia, palpable mass 1 month after first trimester abortion	16	Unilateral adnexectomy	EMA-CO	No

Nakayama et al., 2011 [13]	26	5 months	9,903	Vaginal bleeding	6.4	Unilateral salpingectomy	None	Yes

Rettenmaier et al., 2013 [14]	32	NA	4,759	Abdominal pain	NA	Unilateral salpingectomy	Patient refused	No

Ubayasiri et al., 2010 [15]	36	6 weeks	3,160	Vaginal bleeding	3	Unilateral salpingectomy	MTX	No

Wan et al., 2014 [16]	54	3 months	291,116	Vaginal bleeding	4	Total abdominal hysterectomy and bilateral adnexectomy	5-Fu and KSM	No

NA: not available; hCG: human chorionic gonadotropin; cm: centimeter; MTX: methotrexate; ActD: actinomycin D; EMA-CO: etoposide, methotrexate, actinomycin D, cyclophosphamide, vincristine; 5-FU: 5-fluorouracil; KSM: kengshengmycin.
